# Health and safety management actions to reduce the impact that medication can have on occupational risks in SMEs

**DOI:** 10.3389/fpubh.2026.1887772

**Published:** 2026-09-23

**Authors:** Virginia Herrera-Pérez, María del Carmen Pardo-Ferreira, Francisco Miguel Salguero Caparrós, Juan Carlos Rubio-Romero

**Affiliations:** Department of Business Organisation and Marketing, University of Malaga, Málaga, Spain

**Keywords:** accident rate, health, medication, occupational, occupational risk, safety, small and medium-sized enterprises

## Abstract

**Introduction:**

Occupational risks and accidents are a global concern, so this analysis has been undertaken by a multitude of researchers. However, there is a limited body of research specifically addressing the influence of medication on occupational risks and, more notably, the development of measures aimed at controlling or reducing the impact that such medication may have on workplace safety and health. This scientific gap is even greater among small and medium-sized enterprises (SMEs).

**Methods:**

The general objective of the research presented here is to identify and explore possible health management actions, based on the experience of occupational physicians that could be adopted to reduce the impact of medication on occupational risks in SMEs. To this end, an expert panel was held in May 2023 using the nominal group technique, in which 10 experts in occupational health and safety participated, with open-ended questions, in order to determine these actions.

**Results:**

The main results were as follows: discourage, reduce, and control self-medication by doctors based on a greater knowledge of the problem by both groups; improve information and training for employers, prevention services, and workers on the side effects and impact of drugs on occupational accidents; unify or integrate the medical-occupational history with the general clinical history of the working population; and establish alarm systems in the IT tools of the healthcare systems.

**Discussion:**

The study not only contributes to a better understanding of the occupational risks associated with medication, but also provides practical recommendations to mitigate them. The findings identify a set of recommendations that, according to the experts’ assessment, could help improve preventive management related to medication use in the workplace.

## Highlights

- Self-medication should be reduced through increased awareness of the problem.
- Unify the medical-occupational history with the general clinical history to reduce the impact on medication risks.
- The study highlights the need to establish alarm systems in medical practitioners' software.

## 1 Introduction

Occupational accidents have been approached in several different ways. Thus, the analysis of risks, accident rates, and the main variables involved, including sex, age, material agent, and various deviations, has been undertaken by numerous researchers. Examples include the research conducted by Gambatese et al. (1), López-Arquillos et al. (2), and Gibb et al. (3). These studies have significantly contributed to the understanding of the factors associated with occupational accidents and have provided an empirical basis for the development of occupational health protection programs. However, the organizational characteristics of small and medium-sized enterprises (SMEs)—including limited access to specialized resources, less formalized preventive management systems, and difficulties in accessing advanced health surveillance programs—make it particularly important to develop preventive strategies tailored to this business environment (4, 5).

Other variables, such as the use of drugs or alcohol and their impact on workplace safety, have also been studied by some authors, such as Oswald et al. (6) and Minchin et al. (7). Regarding medication and its effect on occupational risks, there are few published studies, including those carried out by Cockburn et al. (8), Gilmore et al. (9), Kopjar et al. (10), and Oza et al. (11), which observed productivity loss due to the use of drugs or that they could contribute to the risk of accidents in different sectors analyzed. López-Arquillos et al. (12) analyzed the specific risks associated with the construction of structures with formwork and the use of drugs and concluded that it could be an important factor to consider in occupational safety and health, particularly noting that among the trades involved in the construction of structures, “operating and monitoring crane transportation was the highest identified risk activity’ (12). Research has been conducted on the use of medical treatments and their impact on certain risks, such as those related to safety in driving vehicles or even operating machinery (13–15). Healthcare experts acknowledge that medication can contribute to the risk of accidents (12). However, there is no research proposing measures or actions to control or reduce this impact.

This study focuses exclusively on medications used for therapeutic purposes—whether prescribed or taken without a prescription—and does not specifically address the recreational use of illegal drugs or alcohol, except when previous literature identifies them as comparable risk factors.

Therefore, the general objective of the proposed research was to identify and explore possible health management actions, based on the experience of occupational physicians, that could be adopted to reduce the impact of medication on occupational risks in SMEs.

## 2 Medicines and their effects

### 2.1 Medicine concept

To address this issue, it is first necessary to clarify the terminology used in this study. The Royal Spanish Academy defines medication as a substance administered internally or externally to prevent, cure, or alleviate disease and its sequelae (16). However, the distinction between medication and the broader concept of drugs is less straightforward. Gálligo (17) notes that the term “drug” may be used differently depending on the context and that its distinction from medication is not exclusively pharmacological, but may also depend on the purpose and circumstances of use. In this regard, the term “drug of abuse” is commonly used to refer to non-medical substances with psychoactive effects that can be self-administered (17, 18).

Rang and Dale (19), from a pharmacological perspective, define a medicine or drug as a chemical substance that produces a biological effect when administered to a living organism, while acknowledging limitations to this definition. These different perspectives illustrate the conceptual overlap between medication and drugs and the importance of clearly delimiting their use in the present study.

Accordingly, the terms “medication” and “drugs” are used functionally in this study according to their potential effects on workers’ functional capacity and their possible interaction with hazardous work tasks.

### 2.2 Medicines and their classification

Medicines can be classified according to different criteria, including their therapeutic use, pharmacological effects and legal status. For the purposes of this study, the therapeutic and functional characteristics of medicines are more relevant than classifications based solely on their legal status or origin.

The World Health Organization (WHO) provides a classification of essential medicines according to therapeutic groups and priority health conditions (20). This classification includes, among others, medicines affecting the central nervous system, cardiovascular medicines, analgesics, antiallergic medicines, anti-infective medicines, muscle relaxants, and medicines for mental and behavioral disorders. These groups include medicines whose potential effects may be relevant to occupational functioning. This list is presented in Table 1.

**Table 1:** World Health Organization classification of medicines.

Anesthetics
Pain and palliative care medicines
Anti-allergic and medicines used in anaphylaxis
Antidotes and other substances used in poisonings
Anticonvulsants/antiepileptic medicines
Anti-infective medicines
Anti-migraine medicines
Immuno-modulators and antineoplastics
Anti-Parkinson’s medicines
Medicines that affect the blood
Blood derivatives of human origin and plasma substitutes
Cardiovascular medicines
Dermatological medicines (topical)
Diagnostic agents
Antiseptics and disinfectants
Diuretics
Gastrointestinal medicines
Medicines for endocrine disorders
Immunological
Muscle relaxants (peripherally acting) and cholinesterase inhibitors
Reproductive health and perinatal care medicines
Peritoneal dialysis solution
Medicine for mental and behavioral disorders
Medicines acting on the respiratory tract
Solutions for correcting water, electrolyte and acid–base disturbances
Vitamins and minerals
Ear, nose and throat medicines
Medicines for joint diseases
Dental preparations

Source: (20).

However, the WHO classification was not used as an analytical or selection framework for the expert panel. Rather, it provides general conceptual background regarding the diversity of medicines potentially involved in occupational contexts.

### 2.3 The impact of medicines on health and safety

The literature addressing medication use and occupational health and safety encompasses several related but distinct areas. These include occupational risks associated with medication use among workers, risks among professional drivers, risks to patients, occupational exposure of healthcare professionals during the handling or administration of medicines, and risks associated with alcohol and non-legal drugs.

For the purposes of the present study, the primary focus was on the first of these areas: the potential occupational risks associated with medication use among workers. The review of the available literature indicated that this specific topic has received comparatively limited attention. Most published studies have focused on the effects or associations between medication use and occupational accidents, injuries or impaired functioning, while considerably less attention has been devoted to identifying practical preventive and management measures.

This gap provided the rationale for the expert-panel study presented in this manuscript, which aimed to identify potential health and safety management actions to address medication-related occupational risks.

### 2.4 The impact of medicines on occupational risks

As mentioned, research on the use of medications and their impact on occupational risks found in the literature is scarce, and nonexistent are no proposals for action measures to reduce or control this impact. We now present the main studies found in the literature at the time of conducting this study.

Gilmore et al. (9), in a study involving 3,394 individuals with work-related injury or impairment, reported an increased risk associated with the use of some medications, including antihistamines, antibiotics and diabetes medications, although the associations varied according to the type of impairment. Similarly, Pickett et al. (21) identified significant associations between regular use of certain medications and injuries among agricultural workers. Studies by Wadsworth et al. (22, 23) also reported associations between psychotropic medication use, accidents, injuries and cognitive failures, particularly in individuals with additional risk factors or mental health problems. In a sparsely documented publication, Verdoux and Bégaud (24) identified the need to study the impact of psychotropic drugs on the population independent of trials conducted by the industry. Palmer et al. (25) further suggested that both mental health conditions and treatment with psychotropic medication may contribute to a proportion of workplace injuries.

Other studies have examined specific clinical conditions and treatments. Vicente-Herrero et al. (26) highlighted the potential occupational implications of chronic migraine and its therapies, emphasizing the importance of considering both the clinical condition and adverse effects associated with treatment. In addition, Yildirim and Ekmekci (27) examined occupational exposure to pharmaceutical powders, illustrating that medication-related occupational risks may also arise from workplace exposure to hazardous drugs, particularly among healthcare workers. Garbarino et al. (28), in a meta-analysis of 13 studies, reported that although benzodiazepines may be associated with increased rates of health damage, the available evidence was insufficient to support a definitive association.

Of particular relevance to the present research, López-Arquillos et al. (12) investigated the occupational risk associated with different medication families in construction activities using an expert panel. Their findings indicated higher risk values for narcotics, antipsychotics and hypnotics, particularly in relation to activities involving cranes and cutting materials. This study provides a relevant precedent for examining medication-related occupational risks through expert judgement.

Therefore, the use of medications and their impact on safety and health have been well-documented in the literature. In this regard, although various studies have documented the association between certain medications and an increased risk of occupational accidents, there is a notable lack of research aimed at identifying specific preventive measures to manage this risk in the workplace. The available literature has focused primarily on describing the relationship between certain pharmacological groups and occupational accidents, while organizational, training, and healthcare coordination strategies to mitigate these effects have received considerably less attention. This gap is particularly relevant in small and medium-sized enterprises, where preventive resources are often more limited and managing emerging risks presents greater challenges.

## 3 Methodology

To determine the potential actions to be taken to mitigate the impact of different lines of medicine on occupational risks, a panel of experts was held.

The expert panel represents a qualitative methodology that provides a valuable perspective on scientific evidence and experts’ perceptions of its application in a specific context (29). In the setting of the present study, the expert panel was chosen using the Nominal Group Technique (known by its acronym as NGT), which is characterized by facilitating discussion and the achievement of thematic convergence among experts on the research question posed (30). Similarly, the NGT consists of one or two questions posed to participants in advance (31).

### 3.1 Selection of experts

Experts were rigorously selected for this study according to (32, 33). Each candidate is scored based on the scores listed in Table 2.

**Table 2:** Flexible scoring system for the selection of panel members.

Merit or experience	Points
Ph.D.	4
Bachelor’s degree in Medicine (or Master’s degree in Medicine)	4
Member Medical Society	2
Professional experience	1 per year
Article published in medical journal	2 per article
Published book or book chapter	2 per book
Is or has been a member of the company medical service or has worked in a hospital or medical service as an occupational physician.	2

Based on these criteria, we contacted the medical services of five External Prevention Services and two prevention services, explaining the objective of the panel and the profile of the experts we needed for it. We obtained the willingness of 19 doctors to participate in this study. Each expert had to meet at least four of the requirements listed in Table 2 in order to participate in the study. Of the 19 experts who initially volunteered to participate, 13 fulfilled at least four of the predefined selection criteria. Of these 13 eligible candidates, 10 ultimately participated in the expert panel, while three declined participation or were unable to attend the scheduled session. As shown in Table 3, none had a score of less than 36 points.

**Table 3:** Scores of the members of the expert panel.

Merit or experience	Exp. 1	Exp. 2	Exp. 3	Exp. 4	Exp. 5	Exp. 6	Exp. 7	Exp. 8	Exp. 9	Exp. 10
Bachelor’s degree in Medicine (or Master’s degree in Medicine)	4	4	4	4	4	4	4	4	4	4
Professional experience	40	24	40	32	30	25	22	27	30	30
Holds a PhD degree	0	4	0	1	0	0	4	0	0	4
Articles published in scientific journals	24	24	6	10	10	10	6	2	0	36
Author or publisher of a book or book chapter	0	2	2	2	0	2	0	0	0	2
Member Medical Society	0	0	0	0	0	2	2	2	0	2
Has practiced as an occupational physician	2	2	2	2	2	2	2	2	2	2
Total	70	60	54	51	46	45	40	37	36	80

Source: Own elaboration.

Before the meeting began, the experts were informed about the objectives of the research, the exclusively scientific use of the information provided, and the confidential treatment of their responses. Participation was voluntary, and all participants agreed to take part in the study. In this regard, it is indicated that formal ethical approval was not required according to the guidelines of the University where the authors of the study are affiliated due to the observational and consultative nature of the study, and prior verbal informed consent was also obtained from all participants’ experts.

### 3.2 Session development

The expert panel session, conducted using the Nominal Group Technique, was held in May 2023 and brought together the 10 selected experts. The meeting was held in person and lasted approximately 4 h.

Following the protocol outlined by Harvey and Holmes (30) for the implementation of the Nominal Group Technique (NGT) in this study, we proceeded with a brief introduction followed by a statement of the purpose of the session. In the second stage, the moderator posed the research question, instructing the experts to record all their ideas without consulting or discussing them with other participants at this initial stage. Based on the literature review and confirmation of the influence of pharmaceuticals on occupational risks, a single open question was formulated to fulfil the research objective:

How can the impact of drug treatments on occupational risks be improved through health and safety management measures or actions?

This question meets the quality requirements for qualitative research in social sciences and public health given its originality and justification (34).

In the third step, participants were invited to share their ideas, during which the moderator recorded all their contributions in writing using structured notes. Prior verbal informed consent was obtained from all participants before the session, including consent to participate and to have the session recorded.

In the fourth step, a discussion was initiated and detailed transcripts of the answers provided by the experts were taken under the moderation of a member of the research team. In this phase, the moderator should emphasize that the participants did not have to agree with all the ideas listed.

### 3.3 Data analysis

Finally, after compiling all the information received, groupings of similar answers given by the experts were carried out according to the criteria indicated in Table 4.

**Table 4:** Proposals identified, level of convergence, and perceived feasibility.

Proposal	Frequency of mention	Level of convergence	Viability in SMEs
Training and information	High	High	High
Family doctor–work coordination	High	High	Medium
Integration of case histories	Medium	Moderate	Low
Biological controls	Low	Low	Very Low
Preventive culture	High	High	Medium

Authors’ classification developed for the present study, informed by the methodological approach described by Coulter et al. (29). High = ≥7 experts, Medium = 4–6 experts, Low = ≤3 experts, Moderate = some disagreement, and Very Low = divergent opinions.

It was opted not to include a subsequent formal voting, scoring or quantitative prioritization phase. However, the frequency with which each proposal was mentioned by participants was descriptively considered during the analysis. These frequencies were grouped into high, medium and low categories solely for descriptive purposes and were not considered a formal measure of consensus or prioritization.

For clarity, the five categories presented in Table 4 represent broader thematic groupings of the eight specific proposals described in the Results and Discussion. Thus, the numerical difference reflects the level of aggregation and does not indicate a discrepancy in the number of proposals identified.

The classification criteria presented in Table 4 were developed specifically for this study to qualitatively summarize the degree of convergence and perceived feasibility emerging from the expert discussion, drawing on the structured expert-assessment approach described by Coulter et al. (29), but without applying their 1–9 appropriateness-rating scale.

Given the exploratory nature of the research, we opted for an adaptation of the Nominal Group Technique focused on generating and discussing proposals, without including a subsequent phase of voting or quantitative prioritization of those proposals.

As a result of this process, eight major proposals for action and recommendations formulated by experts were identified, which form the basis for the findings presented in the following section. These categories reflect the main areas of intervention identified by the experts to improve the preventive management of the impact of medication on occupational risks, particularly in the context of small and medium-sized enterprises.

## 4 Results and discussion

The following discussion should be interpreted in light of the exploratory qualitative design of this study. The proposed measures represent expert-informed recommendations intended to stimulate future research and preventive practice rather than empirically validated interventions. The results obtained through the expert panel reinforced and expanded previous findings identified in the scientific literature, which already pointed to an association between certain pharmacological treatments and an increased risk of occupational accidents, especially in tasks that require high levels of attention, coordination, or response time (12, 21–23). However, unlike most existing studies that focus on identifying risk, the experts’ contributions allow us to move towards operational proposals for preventive management, an aspect that has been scarcely developed to date.

Below are the main proposals made in response to the question: What measures or actions can be taken to reduce and/or control the impact of medicine consumption on occupational risks? The proposals can be summarized as follows:

*1st Proposal*: *To reduce and control the impact of medication through the measures taken by doctors, and to discourage self-medication by those affected, based on a greater knowledge of the problem by both groups*.

One of the measures or actions that could be carried out to reduce and/or control the impact of the consumption of medicines on occupational risks would be to adjust medication very well by the family doctor/workplace doctor and other specialists, when they have greater knowledge of medicines and their side effects on occupational risks, as indicated by one of the experts *“Medication should be adjusted very well with the family doctor/workplace doctor and other specialists”*. It would be essential to stress the use of medication throughout the occupational physician’s clinical-occupational history, as there is an excessive medicalization of life, which logically has repercussions on occupational risks, as indicated by one expert, among others, who explained that *“the use of medication should be stressed throughout the occupational physician’s clinical-occupational history.”* We must not forget that in many cases, inadequate use in certain pathologies can also produce undesirable side effects (drowsiness, cardiovascular effects, sensory perception deficits, etc.). Consumption should, therefore, be well adjusted, reducing the excessive use of medicines in most cases and adjusting them in fewer cases so that the worker does not under-medicate. This measure is linked to the second proposed measure of information and training, as it would be necessary to start by raising awareness among doctors and healthcare workers in general, so that prior to taking medication, the doctor knows the type of work and the occupational risks to which the patient is exposed in order to prescribe medication that does not affect or increase these risks; in addition, it also considers making it compulsory to inform the medical team when taking any medication, whether or not it is covered by the National Medicines Act in Spain, that is, whether it is classified as natural or chemical.

In addition, it would also be important to discourage the general population from self-medication, which is considered important by experts. This requires a change in the way society views self-medication. In this sense, one of the experts indicated that *“Medicines should not be used by workers without consulting their doctor, because of the possibility of affecting their work and everyone’s safety”*. Another indicated that *“In general, there is an over-medicalization of life, which, logically, has repercussions in the world of work. Many of these medicines, due to inadequate use in certain pathologies, can produce undesirable side effects such as drowsiness, cardiovascular effects, sensory perception deficits, etc.”* Another expert put it this way: *“The fundamental thing would be to be able to de-escalate the excessive use of medicines, but for this to happen, the vision that has been implanted in society with respect to them would have to change.”*

*2nd proposal: Improve information and training for doctors, employers, prevention services and workers on the side effects of medication and its impact on accidents at work*.

This second proposal is closely linked to the previous proposal and is a tool for achieving the objectives of the first proposal. It would consist of establishing clear and sufficient channels of communication and information between the health administration, prevention services, both in-house and external, and the workers, to provide as much information as possible at all levels about the risks inherent in taking medication and its impact on occupational risks. This information and continuous training should highlight the importance and dangers of taking medication without a doctor’s prescription. In this way, adequate training and information could be provided to doctors so that the side effects of medicines and their impact on occupational accidents could be passed on to the working population through primary care and health surveillance, with the aim of generating greater knowledge about medicines and their side effects. This training should be aimed at all health personnel, employers, prevention technicians, and workers, with the aim of influencing the consumption of medication throughout the clinical-occupational history. Thus, one of the experts indicated that *“professionals should be properly informed at the time of obtaining and renewing their license about the list of medicines that can influence their work”*.

These proposals are consistent with the available scientific evidence, which emphasizes that the impact of medication on occupational accidents cannot be analyzed in isolation from the clinical context, type of medicine, and specific working conditions (25, 26). Previous studies have shown that certain psychotropic and sedative medicines are associated with cognitive impairment, accidents, and injuries, especially when other vulnerability factors or a lack of adequate information is present (23, 28). Experts’ insistence on training physicians, workers, and prevention services reinforces the need for a preventive approach based on knowledge rather than indiscriminate restrictions on the use of medicines.

*3rd proposal: Unify or partially integrate the medical-occupational history with the general medical history of the working population and establish alarm systems in the IT tools*.

The unification and/or partial integration of the medical-occupational history with the general clinical history of the working population would be of great help to both the family doctor and the occupational physician to achieve the objectives of the first proposal. This communication should be active between the family doctor and occupational physician to be aware of the actions taken by the worker. In addition, it would be advisable to draw up a list of professions and medicines, and to sensitize occupational physicians to ask patients about their profession so that in the event of a conflict between the profession and its activities and risks and the prescribed medication, the computer system would inform the physician in the form of an alarm of the potential impact. This was expressed by one of the experts as follows: *“The doctors should be made aware of the fact that they should ask patients about their profession. There should be a list of conflicting professions and some form of alarm should be triggered in the computer programs”*. Another expert indicated, *‘The unification and/or partial integration of the medical-occupational history with the general medical history of the working population would be very clarifying for both the family doctor and the occupational physician’.* It should be noted that this result had already been identified in the literature, as in Vicente-Herrero et al. (26).

In this sense, and as studies such as that of Vella et al. (35) point out, clinical information systems based on Artificial Intelligence can help in the identification of risks to occupational safety and health, the prioritization of risks and preventive planning, improving the efficiency and standardization of the medical-occupational history.

However, integrating occupational medical history with general medical records poses significant legal and ethical challenges, particularly in relation to personal data protection, medical confidentiality, and workers’ fundamental rights (36–38). In accordance with current data protection regulations and the principles set out in the international conventions of the International Labor Organization (ILO), any exchange of medical information must be strictly limited to what is necessary for the protection of safety and health, based on the principle of data minimization and accompanied by explicit guarantees of confidentiality and informed consent.

In this sense, rather than a full integration of records, the panel’s findings should be interpreted as a proposal for functional coordination between healthcare professionals aimed exclusively at risk prevention, avoiding indiscriminate access to sensitive clinical information in all cases.

*4th proposal: Carry out specific health examinations and medical checks*.

Another measure or action that could be taken to reduce and/or control the impact of medication consumption on occupational risks, depending on the occupational risks and the effect of medication, is to include specific health tests in the pre-employment health check-up. This occupational health examination should be considered mandatory for jobs that are subject to such occupational risks.

It is also proposed to open up the possibility of carrying out unannounced controls in the workplace, looking for active ingredients in the blood, and testing for the presence of medicines in urine or saliva, similar to those carried out in the field of driving for drugs and alcohol. A basic neurological examination could even be conducted before the start of the working day, as is done in the control of drivers by the General Directorate of Traffic of the Spanish Government, in workplaces where a decrease in the level of consciousness (even if only slight), coordination, visual acuity, and response time could pose a risk to the safety of workers or third parties present in the workplace. This measure would, of course, be subject to the legal possibility of doing so in accordance with applicable regulations. One expert said, *It should be possible to test for the presence of drugs and medicines in urine or saliva*.” Another expert indicated: *“The possibility of carrying out unannounced blood tests for active ingredients would be a recommendable measure.”*

Proposals relating to specific medical checks, including unannounced checks or active ingredient screening tests, should be approached with caution. Although experts propose them from a preventive safety perspective, their implementation could conflict with labor legislation, data protection regulations, and the fundamental rights of workers, particularly the right to privacy, non-discrimination, and medical confidentiality (38).

International evidence and the regulatory frameworks of the International Labor Organization (39) and the World Health Organization (40) emphasize that health surveillance measures must be proportionate to the risk, technically justified, and aimed at adapting to the workplace, never at punishing or excluding the worker. In this regard, these proposals should be understood only as possible exceptional measures, subject to a clear legal basis, collective bargaining, and strict ethical guarantees, or reinterpreted as mechanisms for functional risk assessment rather than as punitive control instruments.

It should also be noted that the proposals regarding unannounced testing for the presence of medications, detection of active ingredients, or functional assessments were put forward only by some of the participating experts and received low levels of convergence and feasibility. Therefore, they should not be interpreted as established recommendations, but rather as potential avenues for consideration in specific high-risk contexts.

*5th Proposal: Adapt the above training and information measures to the characteristics of SMEs and their employees*.

One of the necessary measures and/or actions to be developed, especially aimed at SMEs owing to their higher accident rate, on the issue under study would be that information and training measures on the problem under analysis, both for employers and for all workers, should be adapted to the characteristics of SMEs and their workers. One expert indicated that: “*Information and training measures on the prevention of occupational risks should be adopted, both for employers and for all workers, adapted to the characteristics of the SME and its workers*’.

In the context of SMEs, characterized by limited resources and reduced access to specialized occupational health services (5), experts agree that training and coordination measures are significantly more viable than strategies based on intensive medical monitoring.

*6th Proposal: To increase the awareness of the different medical groups since, although there is a level of perception of the problem in the medical profession, this is lower among specialists and somewhat higher among family doctors and occupational physicians*.

In general, the perception is greater in those groups in which there is some subsidiary responsibility for possible side effects associated with occupational risks. Thus, there is a perception that specialist doctors, in general psychiatrists and neurologists, among others, show less awareness, while family doctors are more sensitive, and in the end, occupational doctors tend to manage this problem with “greater awareness.” Thus, it is less taken into account than necessary because not all workers are reached, nor is compliance monitored regularly and exhaustively. One of the experts stated the following: *“Yes, I believe that medical professionals take it into account, but there are branches of medicine, such as the occupational medicine branch, which are more knowledgeable in order to be able to issue the aptitude and, if necessary, the absolute or partial restrictions to these risks.”* Another practitioner stated, ‘*In general, they take into account those groups in which there is some subsidiary liability derived from possible secondary effects associated with occupational risks. In such a way that I perceive, from my own experience, that specialist doctors in general (psychiatrists, neurologists, ...) show little concern, and family doctors somewhat more, with the occupational doctors being the ones who tend to manage this problem with ‘greater awareness.”*

*7th Proposal: Increasing the preventive culture*.

It is argued that the biggest barrier is the scarce or non-existent “preventive” awareness of all the agents involved in the regulatory development included in the EU Framework Directive on Occupational Safety and Health, both in the company and official bodies, as well as in the workers themselves. This preventive culture must reach all of them. However, there are many shortcomings in this respect. This was expressed by one of the experts as follows: *“The preventive culture has to reach the whole population, both employers and workers. There are many shortcomings in this respect”*. Another of the experts indicated: *“There is no real preventive culture and the scarce one that exists stems from the “punitive obligatory nature” of non-compliance with legislation*”.

*8th Proposal: To reduce the under-diagnosis of certain pathologies*.

Another proposal made by the experts can be summarized in the words of one of them: *“the under-diagnosis of diseases such as sleep apnoea, diabetes (poorly controlled), alcohol and other drug use, and/or mental health problems are barriers to the implementation of measures. In the case of mental health problems, moreover, the difficulty is that they are not reported because of shame and fear of being stigmatized”*.

## 5 Conclusion

This study explored in depth the measures required to mitigate or reduce the impact of medication on occupational risk. Based on these findings, the main proposals identified by the expert panel can be summarized along several key lines. First, there is a need to reduce and control this impact through appropriate medical management while discouraging self-medication by fostering greater awareness of the issue among both healthcare professionals and workers. Second, it is essential to strengthen the information and training for physicians, employers, prevention services, and workers regarding the side effects of medications and their influence on occupational accidents. In addition, partial integration or unification of occupational medical records with general clinical histories is recommended, along with the incorporation of alert systems within electronic health tools. The implementation of specific health examinations and targeted medical controls was also highlighted. In the case of small- and medium-sized enterprises (SMEs), these training and information measures should be specifically adapted to their structural and workforce characteristics. These proposals should be interpreted as recommendations based on expert opinion that can serve as a foundation for future research and preventive practices.

The findings suggest a certain level of awareness within the medical profession regarding the extent to which the impact of medication on occupational risks is currently considered. However, this awareness varies by specialty; it is generally lower among specialists and somewhat higher among general practitioners and occupational physicians. However, it is acknowledged that such awareness does not reach all workers and is not subject to systematic and comprehensive monitoring.

Finally, with respect to the main barriers to improving the management of medication-related occupational risks, the lack of a well-established preventive culture was identified as the most significant limiting factor.

All proposals identified must be interpreted within an ethical and legal framework that guarantees rights, in which occupational health and safety do not conflict with the fundamental rights of workers, but rather reinforce them mutually, especially in the context of SMEs.

### 5.1 Future lines of research

Future research should evaluate the implementation and effectiveness of the measures identified by experts in SMEs across various sectors, as well as develop specific tools to systematically assess and manage occupational risks associated with medication use.

Furthermore, future studies could employ more specific criteria for selecting experts, focusing on particular sectors or professionals with direct experience in managing occupational incidents related to medication use.

## Data Availability

The original contributions presented in the study are included in the article/supplementary material, further inquiries can be directed to the corresponding author/s.
